# Motivations, Decision-Making, and Barriers to Treatment Innovation Adoption: A Mixed-Methods Study of Addiction Treatment Executives

**DOI:** 10.21203/rs.3.rs-10541589/v1

**Published:** 2026-08-03

**Authors:** Tyler Wray, Erik Ocean, Sonal Sharma

**Affiliations:** Brown University; Brown University; Brown University

**Keywords:** Substance use disorder treatment, innovation adoption, implementation science, addiction treatment organizations, organizational decision-making

## Abstract

**Introduction:**

Evidence-based treatments for substance use disorders (SUDs) are widely available, yet many facilities struggle to adopt and implement them at scale. Little is known about how facility leaders decide to adopt treatment innovations or what motivates and impedes those decisions. In this study, we explored motivations, decision-making processes, and barriers to innovation adoption among addiction treatment executives.

**Methods:**

We conducted an exploratory sequential mixed-methods study with 12 executives and clinical directors of addiction treatment centers in New England. Semi-structured qualitative interviews were analyzed using codebook thematic analysis. Findings informed a follow-up quantitative survey administered to the same participants and analyzed descriptively.

**Results:**

Treatment facility leaders held generally positive attitudes toward innovation and often follow a relatively deliberate adoption process. Improving patient outcomes and retention was the most salient motivation across both qualitative and quantitative data, but many described patient referrals, financial sustainability, and staff wellbeing as related rather than competing priorities. Innovation ideas most often originated from clinical staff, peer networks, and conferences. Peer-reviewed journals were among the lowest-rated discovery sources. Regarding barriers to adopting treatment innovations, roughly a third of participants rated trialability as an important factor in adoption decisions, and piloting before full implementation was common but not universal. On surveys, the need to add staff, workflow compatibility, and mission alignment were the highest-rated inner setting barriers, while staff training needs were among the least important.

**Conclusions:**

These findings suggest that barriers to adoption arise less from facility leaders’ attitudes and more from organizational and operational constraints. Interventions that align with existing workflows can be tested on a small scale. Leveraging clinical and professional networks for dissemination may be more likely to achieve widespread implementation.

## 1. Introduction

A wide range of effective behavioral and pharmacological therapies are available for treating substance use disorders (SUDs) (Volkow & Blanco, 2023). However, many patients with SUD who present for treatment in the US still do not receive evidence-based treatment (Substance Abuse and Mental Health Services Administration (US) & Office of the Surgeon General (US), 2016). According to the 2022 National Survey on Drug Use and Health, of the 13 million US adults who received SUD treatment, only 30% received medications to address problems with alcohol or opioids (Substance Abuse and Mental Health Services Administration, 2023). Likewise, while many facilities report providing evidence-based behavioral therapies for SUD, what is actually delivered in practice often lacks the fidelity to achieve similar outcomes as those observed in research settings (Addiction Medicine, 2012; Carroll & Rounsaville, 2007). These findings suggest that many SUD treatment facilities are not providing the latest evidence-based care, either because these latest treatments are not adopted at all or because they are implemented incompletely.

A substantial body of research has sought to identify organizational and workforce-related determinants of evidence-based practice (EBP) adoption in SUD treatment, much of it focused on behavioral innovations such as contingency management (CM). This research has shown that, at the organization-level, adoption was more likely when treatment philosophy was compatible with the innovation, when facilities served populations that were subject to external mandates (e.g., drug court referrals), when they had prior involvement in clinical research networks, and when clinics were non-profit or publicly funded (Bride et al., 2011; Ducharme et al., 2007). At the workforce level, higher staff education and positive attitudes toward manualized treatment predicted adoption, while competing clinical demands, staff turnover, complicated certification processes, and client or staff resistance were key barriers (Amodeo et al., 2011; Becker et al., 2023).

Similar studies have examined determinants of pharmacological innovation adoption. These studies have shown that facilities were more likely to adopt medication programs like buprenorphine if they already had resources that the innovation required, such as having physicians on staff already, experience with other SUD treatment medications, administrative experience, and managed care involvement, or if they had previous clinical trial network experience (Abraham & Roman, 2010; Ducharme et al., 2007; Roman & Johnson, 2002). Findings on facility size have been mixed and appear more strongly associated for pharmacological innovations than behavioral ones, suggesting that determinants of adoption meaningfully differ by innovation type (Abraham & Roman, 2010; Bride et al., 2011; Roman & Johnson, 2002). Together, this research shows that a variety of workforce and organizational factors affect the adoption of *specific* treatment innovations (see Table 1 for a list of determinants), but because many findings are intervention-specific, it remains difficult to identify a set of core predictors that generalize to many different types of innovations.

Whether any factors consistently predict adoption across innovation types remains an open question. Knudsen and Roman (2004) found that facilities that had adopted a greater number of innovations frequently engaged in “environmental scanning,” which refers to encouraging staff to participate in professional development, professional organizations, and review research (Knudsen & Roman, 2004). Facilities that collected satisfaction data from payers and referral sources and employed a higher percentage of staff with Master’s degrees also had adopted more innovations. Higher levels of organizational stress, client complexity, and a longer organizational history were all significantly associated with reporting more barriers to implementation (Lundgren et al., 2012). Motivation to adopt EBPs was generally high, especially for behavioral interventions, but clinicians that identified with 12-step approaches to treatment also reported lower readiness to adopt medications or non-12-step behavioral interventions (McGovern et al., 2004).

Most of this research has relied on quantitative, facility-level data. Incorporating qualitative data could add richness and surface the full range of factors that could play a role. Understanding adoption barriers from the perspective of facility directors could be especially useful, given their role as decision-makers.

Few studies have been uniquely focused on understanding the decision-making process that treatment centers engage in when considering adoption of treatment innovations. Several frameworks have been developed to describe how organizations change, such as the Exploration, Preparation, Implementation, Sustainment (EPIS) (Aarons et al., 2011) or Organizational Readiness to Change models (Saldana et al., 2007), but few empirical studies have explored how addiction treatment facilities actually consider and decide to adopt specific treatment innovations. How do they learn of new approaches to treatment? How are they shared with the executive team? Who plays a role in decision-making?

Similarly, the *motivations* behind adoption decisions have received little direct study. Knudsen and Roman (2015) explored the characteristics of innovations that made them most appealing to treatment facility leaders and found that, consistent with other studies (McGovern et al., 2004), interest in adoption was strongly associated with the facility’s treatment philosophy (Knudsen & Roman, 2015). Leaders of 12-step oriented facilities, for example, were less likely to adopt pharmacological innovations (Knudsen & Roman, 2015). Leaders also expressed high interest in innovations that could reduce dropout rates or improve their facility’s reputation among referral sources (e.g., courts, physicians). Factors such as increased revenue or organizational growth were considered less important. External mandates such as accreditation standards have also been identified as drivers (Knudsen & Roman, 2004). Still, few studies have directly elicited the motivations that drive leadership’s innovation decisions.

To address these gaps, we conducted an exploratory sequential mixed methods study of addiction treatment center executives and leadership (chief executive officers, executive directors, clinical directors) to address three key questions: (1) what motivates executives to adopt innovative treatment options? (2) what are the key elements of the decision-making process they go through when deciding to adopt new treatment options? (3) what barriers do they perceive as most important to adopting treatment innovations? We first conducted individual, semi-structured qualitative interviews with participants to elicit themes related to each of these questions. We then used these themes to develop and administer a follow-up quantitative survey to the same participants, which allowed us to validate and prioritize findings across the full sample.

## 2. Methods

### 2.1 Participants

We recruited 12 executives or clinical leaders of addiction treatment centers in the New England area through direct outreach via email or on LinkedIn. Participants were eligible if they (1) held positions such as chief executive officer, executive director, or clinical director, (2) at an organization that publicly offers addiction treatment services. Recruitment was purposive to ensure a mixture of leaders of facilities of various sizes and various levels of care (e.g., detox, inpatient, residential, outpatient-only). Recruitment continued until saturation was reached, i.e., new interviews no longer introduced novel themes or required revision of the codebook. To protect confidentiality while preserving role context, participants are identified by role-based codes throughout: EP (executive participant) and CP (clinical participant).

### 2.2 Procedures

Eligible participants scheduled interviews using an online scheduling system. Participants were not formally required to document their informed consent because they were considered “key informants” being asked to provide their professional opinions and thus were not considered to be human subjects. However, our procedures adhered to all standards that are typically applied to research involving human subjects. At the beginning of each interview, participants were provided a brief description of what the study would involve, the right to withdraw at any time without penalty, confidentiality, and potential risks. All interviews were conducted by the first author (TBW) via videoconferencing and were recorded for later transcription. Interviews typically lasted about 60 minutes and were conducted between August 2025 and December 2025. Qualitative findings informed the development of a follow-up survey, with items and response options derived directly from interview themes and refined through multiple rounds of author review. Survey responses were collected from February 2026 to April 2026. Participants were paid $200 for completing the qualitative interview and $150 for completing the online survey. These procedures were not reviewed by an Institutional Review Board, as participants were considered “key informants” because they were exclusively asked about their perspectives in their professional capacities.

### 2.3 Measures

#### 2.3.1 Qualitative interviews

Individual, semi-structured interviews elicited broad themes about participants’ primary motivations for considering innovations in their center’s treatment offerings, decision-making processes, and barriers to adoption. Participants were first asked to recall a time when they considered adopting a new treatment offering or changing their treatment offerings, using this situation as a reference throughout. We defined an “innovation in treatment offerings” as any program, service, or approach to care intended to have therapeutic value for patients and new for the facility, such as a counseling approach (e.g., mindfulness, acceptance and commitment therapy), complementary or alternative service (e.g., yoga, music therapy), or medication program (e.g., buprenorphine). Prompts addressed motivations for adoption (including the roles cost, research evidence, and regulatory factors), the decision-making progress (e.g., discovery, stakeholder involvement, financial analysis), and the most significant barriers and facilitators to adoption.

#### 2.3.2 Follow-up survey

Following qualitative analysis, we developed a follow-up survey to quantify and validate themes identified in interviews. Items and response options were drawn directly from interview themes and covered motivations for adoption, discovery channels, staff latitude for innovation, decision-making involvement, financial analysis practices, piloting practices, and innovation characteristics. For several key items, participants were asked to assign a percentage to each response option. This approach captured relative importance while avoiding the common problem of participants rating all response options highly. To characterize participants’ overall attitudes toward innovation and their perceptions of their peers’ and organizations’ attitudes, we also collected the Attitudes Toward Innovation Survey (ATIS), a well-validated 24-item scale (Sibley et al., 2023).

### 2.4 Data analysis

#### 2.4.1 Qualitative data analysis.

We used codebook thematic analysis (Braun & Clarke, 2023), taking a hybrid approach: codes and themes were generated inductively from the data, but EPIS and CFIR served as sensitizing concepts that shaped how we interpreted and organized what we found. We aimed for interpretive themes, seeking to capture meaning and theoretical constructs rather than topic summaries. An initial coding template derived from the interview guide and team domain knowledge was iteratively revised through contact with the data. Three expert coders constructed an initial codebook before systematically coding. The team coded the first three transcripts collaboratively to calibrate shared understanding before two coders independently coded each remaining transcript. Coders met regularly to resolve interpretive questions and collaboratively refine the codebook by adding, splitting, merging, or refining codes, consistent with the constant comparative method (Glaser & Strauss, 2017). A third coder reviewed all disagreements and established a final, reconciled transcript.

Following coding, thematic synthesis developed interpretive themes from patterns across codes. We applied an analytical framework structured along two dimensions: innovation stage and stakeholder role, with stakeholders and motivational drivers further organized by inner versus outer context. The stage dimension draws on the EPIS framework, which divides the adoption of evidence-based practices into the phases Exploration, Preparation, Implementation, and Sustainment (Aarons et al., 2011). The inner/outer distinction draws on CFIR, which differentiates organizational-level factors (inner setting) from environmental, regulatory, and community-level factors (outer setting), and also informed our attention to characteristics of the innovation itself (the “innovation” domain) (Damschroder et al., 2022). This framework was applied after thematic synthesis to illuminate and situate findings, rather than impose an external structure on the data.

#### 2.4.2 Quantitative data analysis.

Follow-up survey data were analyzed descriptively using appropriate summary statistics. All analyses were conducted in StataSE 19 (College Station, TX).

## 3. Results

### 3.1 Participant & facility information

Participant and facility characteristics are shown in Tables 2 and 3. A majority of participants were middle aged, male (84.6%), non-Hispanic White, and had graduate degrees. Two-thirds were chief executives, executive directors, or presidents of their organizations, while the remaining third were clinical directors. Participants largely represented for-profit, free-standing treatment centers. Around half offered treatment services beyond outpatient, such as inpatient, residential, or recovery residences. Size varied considerably, with facilities serving a median of 1,200 patients (*IQR* = 5,200). Alcohol was the primary substance for nearly half of the patients served, opioids for nearly a third, and stimulants, cannabis, and multiple drugs for the remainder. Participants had attitudes toward innovation that were slightly above neutral on average on the ATIS (*M* = 3.3, *SD* = 0.3), while rating their colleagues’ attitudes as slightly below neutral (*M* = 2.9, *SD* = 0.6), and their organizations’ attitudes as neutral (*M* = 3.0, *SD* = 0.6).

### 3.2 What motivations for adopting treatment innovations are important?

Five key themes emerged from participants’ responses about motivations for innovation adoption, though participants frequently noted these themes frequently overlapped and were often mutually reinforcing.

#### 3.2.1 Theme: Improving patient outcomes and retention

Across all interviews, improving the health outcomes of their patients was core to their motivations: “Everything we do is around, ‘What does the patient population we serve need that we don’t offer? And is there an opportunity for us to do that?’” (EP7). Several mentioned that organizational goals, such as financial sustainability and growth, are effects of ensuring optimal care.

Participants described client retention and outcomes as closely related, with many innovations targeting both simultaneously. Many innovations discussed were motivated by directly improving patient outcomes (e.g. a new community health worker step-down program for patients who have completed outpatient care but still need support), but many others were focused on improving retention or preventing patients from leaving early. Strategies for preventing patients from leaving “Against Medical Advice” (AMA), were among the most frequently mentioned types of innovation. Participants described a range of approaches to address this issue, from efforts to involve family or community members to structured weekend programming (e.g., yoga), entertainment and amenities:

“The patient needs to buy into ‘the longer I can get treatment for, the greater my successful outcome is going to be’. But the patient needs to be ‘entertained’ during that period of time. And we do a lot of different things to keep the patient entertained”

(EP1).

#### 3.2.2 Theme: Improving referral relationships and reputation

External referral relationships functioned as both a motivation for and source of innovation. Maintaining strong relationships with referral partners such as departments of corrections, courts, emergency departments, and primary care providers depended in part on being seen as offering the latest innovations in care. Continuing to receive referrals from partners was directly tied to the facility’s financial well-being and often outperformed general marketing in terms of new patient acquisition. Some participants gave examples of referral partners directly encouraging innovation by requesting that referred clients receive specific treatment. Adding medication options at the request of a community partner, one participant reported, “blew the doors off” their referral volume (EP4). Some participants mentioned that adopting innovative treatment options could sometimes increase referrals from other treatment centers as well, who would route clients to facilities that offered specialized services:

I think especially with a lot of the other facilities in the area that have similar offerings, but not exactly the same, that’s where we see the benefit of some of those more unique offerings because our admissions team works really closely with all these other programs… They’ll know, if someone [is] specifically looking for a lot of creative arts integration, we're the only one. So they know that they can send them to us and vice versa.

(CP10)

Market differentiation was a closely related motivation:

“I think that… [at the] department level, there is a hunger for that stuff because that’s what keeps us competitive as a place where people will seek treatment, right? So there is a desire to keep up with other systems who are starting to offer like ketamine treatment, S-ketamine and having TMS [transcranial magnetic stimulation].”

(CP11)

Reputation among patients and the broader community also mattered:

“We pay attention to our reputation online and look at things like Google reviews and Shatterproof… we know for all of our community relationships that if people don’t feel like we're doing a good job, they will refer elsewhere. And why not? They should.”

(CP8)

#### 3.2.3 Theme: Financial sustainability

Nearly all participants described clinical success and financial health as fundamentally aligned. One executive called it a “spiritual law” of the sector: good patient outcomes produce good financials (EP3). Financial motivations nonetheless shaped which innovations were pursued. Participants described piloting innovations, often without immediate payoff, like EP6: “Every successful program that we delivered usually starts for free. So we kind of eat… the cost of innovation first” (EP6). Other participants noted that innovations must eventually generate returns through higher reimbursement rates, larger payer contracts or improved retention. Non-profits could sustain unprofitable programs indefinitely, as long as the unmet need was significant and the losses could be offset by surpluses elsewhere.

Participants also described a range of billing and payer strategies that motivated innovation. “Expanding the payer mix” (e.g., increasing commercial contracts, out-of-network coverage, Medicare eligibility) often opened access to new patient populations. Formalizing services that had previously been offered informally created new billable products. Facilities preferred bundled reimbursement codes over ancillary billing, and negotiating these codes with insurers was itself a driver of innovation, since new service models required new reimbursement structures to be financially viable.

#### 3.2.4 Theme: Organizational mission

Participants described patient welfare as a core organizational value. Several participants self-identified as being in recovery themselves, and noted that many of their staff were as well: “It’s an altruistic segment of healthcare. And I would say… two thirds of our employees are in recovery. And probably closer to 90 percent of the peer coaches or more are in recovery” (EP2). This gave many staff a deep understanding of both the patient population and the challenges they face. One executive noted that their own experience as a patient at multiple treatment centers motivated them to innovate around AMA prevention and retention.

#### 3.2.5 Theme: Staff fulfillment and morale

Participants described supporting staff clinical interests as both an organizational value and a practical strategy. Executives described approving clinician-led pilots and training not only for patient care benefit but because supporting clinicians' own clinical interests reduced burnout and gave staff a sense of ownership over the treatment they delivered. Several noted that leaders with personal recovery identities made this dynamic easier to sustain, since clinical directors could propose and pilot innovations knowing there was a genuine organizational interest in improving care.

#### 3.2.6 Quantitative results

Survey results about participants’ most important motivations for innovating were consistent with qualitative results in some ways, but inconsistent in others (see Table 4). Consistent with qualitative findings, most participants rated “to improve [their] patients’ well-being” highest. “Attract[ing] move patients to [their facilities]” was the second most highly rated, but in contrast, “to attract more referrals (from doctors, courts, insurers) was among the lowest-rated motivations. Improving staff morale and avoiding burnout was highly rated despite rarely being raised in interviews. Finally, complying with accreditation standards was rated highly, despite not being raised in interviews. See Table 4 for descriptive statistics by item.

Also consistent with qualitative findings, participants rated promises to “increase long-term recovery rates” as one of the most appealing claimed benefits of potential treatment innovations, closely followed by “improves engagement and retention in treatment.” More operational benefits such as reducing costs, increasing census stability and improving operational efficiency, were not rated as highly. See Supplementary Table 1.

### 3.3 What are the key parts of the decision-making process when deciding to adopt new treatment options?

Participants’ descriptions of their innovation processes mapped onto the four phases of the EPIS framework (Aarons et al., 2011): Exploration, Preparation, Implementation, and Sustainment. Key themes are identified and described below, categorized by phase.

#### 3.3.1 Theme: Exploration: Problem solving

Several participants traced the beginning of their innovation process to encountering problems at their facilities and searching for solutions. Appointment no-shows, high rates of premature discharge, and care transitions that patients fell through all functioned as triggers for exploration. However, a few also reported being more opportunity-driven, remaining open to innovations they heard about through others in the field or consulting regularly with clinical staff about potential improvements. Some also reported explicitly carving out time for staff to explore potential improvements or to try new things. Both executives and clinical staff also noted regularly engaging with peers through conferences, trainings, or workshops as a regular source of new ideas. Commercial vendors approaching participants with “sales pitches” were also mentioned as a channel for discovery.

#### 3.3.2 Theme: Exploration: Constraints and operational bandwidth

Even when participants were motivated to explore new approaches, day-to-day clinical demands crowded out the capacity to do so. One participant said day-to-day operations made it hard “to zoom out and say, big picture… ‘how do we improve clinical care?’… I don’t think many people have access [to] the bandwidth always to do that, and I don’t think it’s for lack of wanting that, I just think it gets forgotten a little bit” (CP11). Participants from facilities without dedicated time for innovation review described becoming more dependent on external sources to initiate exploration. For example, one participant described how their facility began exploring extended-release, injectable naltrexone (Sublocade^®^) after the Department of Corrections began requesting that their cases receive it to reduce diversion risk. Participants from organizations with multiple facilities also noted sharing and reviewing “implementation playbooks” from other sites.

#### 3.3.3 Theme: Preparation: Pre-adoption scrutiny

Participants reported that their facilities have done a range of things to help them weigh whether to pursue a given treatment innovation, including financial analyses, discussions with various stakeholders, and pilots. However, several noted that the “intensity” of each of these activities varies depending on the complexity of the innovation. For example, starting a new type of counseling group or offering a new complementary health service (e.g., a yoga class) were often adopted with little formal review or analysis, whereas more complex innovations (e.g., medication services) might warrant auditing personnel and training needs, physical space requirements, or even reviewing the facility’s alignment with Drug Enforcement Agency criteria. One participant also said that the extent of analyses and preparation also may scale with organization size: “We've always been a pretty small team and it’s kind of been like, ‘That sounds like a good idea. Let’s do it.’ … And so [as we grow] that’s getting a little bit more formal” (CP10).

#### 3.3.4 Theme: Preparation: Financial vetting

Many participants characterized their approach to financial vetting as informal, with one describing it as “napkin math” rather than formal projections. However, participants varied in how much financial scrutiny they applied, with some oriented toward projected return and others oriented simply toward sufficiency, where costs would be covered. Key considerations included whether demand existed, whether the innovation fit with existing infrastructure (e.g., space), and whether services were billable. Nonprofit participants in particular noted more flexibility here: one participant described operating a children’s detox program at a loss for 10 years because it was an unmet community need (EP7). Still, establishing pathways to pay for any new services was a common constraint, especially if new programs required novel billing structures. Some noted preferring innovations that could potentially be included in bundled care contracts, as opposed to fee-for-service.

#### 3.3.5 Theme: Implementation: Piloting and iteration

Piloting before full implementation was common. One participant described their facility’s standard approach as “experiment[ing] with a lot of stuff and throw[ing] stuff against the wall to see if it works or doesn’t work” (EP1). Another called it “dipping [their] toe in the water” (EP2). From there, implementation was iterative: programs were modified, tailored to specific facilities, or temporarily suspended based on patient engagement.

#### 3.3.6 Theme: Implementation: Clinician champions

New treatment innovations were often led or “championed” by clinical staff with a particular interest in the area. Several organizations institutionalized this by maintaining open or “free” curriculum slots in inpatient and residential programs, giving clinicians a low-stakes channel to pilot ideas that emerged directly from patient contact. This was described as a way to both surface good innovations and retain staff.

#### 3.3.7 Theme: Sustainment: Balancing clinical outcomes with financial viability

Participants reported deciding to sustain or cancel programs based on fidelity, utilization, impact on retention, and financial viability. Several said they would sustain programs that improved outcomes even without direct revenue, as long as losses could be offset elsewhere. Family intervention programs were an example of this, sustained for their association with reduced AMA rates and longer lengths of stay despite generating no direct revenue. Some facilities also reported using validated clinical outcome measures such as PHQ-9 and GAD-7 to evaluate program impact, though this was not the norm. Other participants prioritized financial viability, noting that they “can’t do things just because,” and that every sustained investment “had to demonstrably drive the business forward” (EP2). Successful pilots were formalized by integrating them into rotating treatment curricula, developing written procedures, and uploading materials to shared platforms to ensure consistent delivery across staff and, for multi-site organizations, across facilities (CP10, EP12).

#### 3.3.8 Quantitative results

Survey results largely echoed qualitative findings. Participants most often reported first hearing about innovations through clinical staff, recommendations from peer organizations or professional networks, or at conferences. Peer-reviewed journal articles were among the lowest-rated sources (see Supplementary Table 2). Nearly half (46%) reported maintaining a designated time for discussing innovations. Most also reported that various stakeholder groups (e.g., administrative staff, patients, family members, finance, legal, and external advisors) were at least consulted when making decisions about an innovation, but most (55%) directly involved clinical staff in final decisions about adoption, and nearly half (46%) reported that the final say was with their board of directors. See Supplementary Table 3.

Nearly three quarters of participants (73%) reported conducting formal financial analyses at least “sometimes,” in contrast with the informal “napkin math” described in interviews. Of those, only one said this involved informal discussion, rather than quantitative analysis. Half (50%) reported formal financial modeling, while the remaining participants reported rough estimates (25%) or basic spreadsheet projections (25%). The most common elements included were start-up costs (82%), operating costs (82%), and staffing and training needs (82%). Fewer participants reported including break-even analyses (46%) or multi-year projections (36%).

Roughly half of participants (55%) reported piloting new treatment programs before fully implementing them, but about a third (33%) reported rarely doing so. The outcomes participants most often wanted to evaluate from a pilot, on a 0 (*not at all important*) to 3 (*extremely important*) scale, were retention in treatment (*M* = 2.7, *SD* = 0.5), successful reimbursement for services (*M* = 2.3, *SD* = 0.9), and overall feasibility (*M* = 2.5, *SD* = 0.5). However, all outcomes were rated as at least “quite important” (see Supplementary Table 4).

### 3.4 What barriers do treatment facility executives believe most impede the adoption of treatment innovations?

To organize barrier themes, we used the Consolidated Framework for Implementation Research (CFIR) 2.0 (Damschroder et al., 2022) to guide our interview and qualitative data analysis. We focused on the innovation, inner setting, and outer setting domains, which we believed exerted the most immediate influence on adoption.

#### 3.4.1 Theme: Innovation domain: Trialability

Several participants described preferring to pilot programs before making any permanent commitment, and the ability to reverse course if a program failed was an important condition for being willing to try at all. One executive framed this as a matter of minimizing risk and ego simultaneously: “minimize the risk… minimize the investment… and then minimize your ego so that when it doesn’t work, you could admit it and pull back” (EP3). Clinical directors used open curriculum slots and designated test sites to try new adjunctive therapies knowing that low attendance or poor reception was sufficient reason to stop: “if something kind of peters out like that, then we take a break from it, try something else” (CP10).

#### 3.4.2 Theme: Innovation domain: Relative priority

Treatment innovations were sometimes seen as ancillary or separate from the “real” treatment activities. For example, one participant noted that executives were “hungry” for treatment innovations at their organization, but that “cutting edge things feel like a little esoteric to like what we're trying to do… we're just trying to get these folks through the week” (CP11). Participants reported that immediate clinical demands often crowded out the time and space to review innovation opportunities. One clinical executive said that staff perpetually “putting out fires” made it difficult to ask them to step back and ask how care could be improved: “I don’t think many people have access to the bandwidth always to do that and I don’t think it’s for lack of wanting. I just think it gets forgotten a little bit” (CP11). Even when staff had the skills to implement something new, competing demands could make it impossible: one clinical director noted that therapists “were so overloaded with massive case loads that there wasn’t a way to touch it” (CP9). Participants also noted that innovation could sometimes be a source of further stress and contribute to burnout.

#### 3.4.3 Theme: Inner setting domain: The culture or treatment philosophy of the organization or staff

Several participants noted that philosophical conflicts between a proposed innovation and the staff, leadership, or facility's treatment approach could be a barrier. One participant said that their facility had an abstinence-focused mission for over 60 years that categorically blocked medication-assisted treatment until leadership said: “Okay, well then you better cut your revenue in half. Because we're gonna lose a lot of grants” (EP3). Philosophical resistance sometimes came from a vocal minority of staff rather than formal organizational policy. They also said that this resistance could be tacit, with unsupportive staff stalling innovations that had already been approved.

#### 3.4.4 Theme: Inner setting domain: Complex bureaucracy

Participants reported that lengthy internal approval chains often added months to implementing a new program. In large systems especially, the approval process required sign-off from multiple layers of leadership before a new program could launch. One participant said that the pace of innovation in large systems is constrained less by resistance than by logistics: “what gets in the way of things is quite literally like time to have the meetings sometimes… making sure the right people can say yes and get approval can just take time in a big system” (CP11). Participants also mentioned upfront costs and the need for additional personnel or specialized training.

#### 3.4.5 Theme: Outer setting domain: Regulatory requirements (and conflicts between them)

Regulatory barriers at the the federal and state levels created friction around adopting new treatments, sometimes because they were not aligned with each other. One participant described Ohio's restrictive stance toward methadone as an example of how state-level policy could effectively remove an evidence-based tool from the table entirely: “Ohio hates methadone. They make it absolutely impossible unless you're a nonprofit that’s basically a methadone clinic. You can’t get a license. It is an effective tool that the legislation here is literally taken off the table” (EP3). At the same time, federal regulations were pushing broad adoption of medication-assisted treatment (MAT). Even when regulations were not a barrier, the approval process could be, and the unpredictability of timelines made planning difficult even when the approval was expected. Participants described it as “not a complicated process… just a long process” (EP4).

#### 3.4.6 Theme: Outer setting domain: Reimbursement negotiations.

Without established billing codes, facilities had no choice but to negotiate reimbursement for novel services payer by payer: “we know that each one is going to be like starting from scratch each time” (EP5). Even with a recognized billing code, these negotiations typically took 6–12 months. The process was described as relational and difficult to systematize, and outcome data carried little weight: “Never once has one payer asked us to see any of our outcome data. They could care less” (EP5). For patients covered by Medicaid-managed plans, getting a novel service reimbursed typically requires federal CMS action, creating a significant bottleneck.

#### 3.4.7 Theme: Individuals domain: Patients are sensitive to even small frictions.

Participants pointed out that clients often seek treatment in moments of crisis (“nobody comes there on a winning streak…You just gotta love them through it,” EP3), making them especially sensitive to minor frictions:

“People seeking healthcare services are not typically flexible, understanding, or in the right mind. And now you're talking about a population that’s in crisis and already not really all in with wanting to seek care. And so the littlest technology barrier will be used as a reason to not seek care.”

(EP2)

#### 3.4.8 Theme: Individuals domain: Staff skepticism.

Participants reported that staff could be hesitant about workflow changes, particularly those coming from executives, shaped in part by prior experience with technology initiatives that overpromised and underdelivered. One participant likened it to research fatigue in under-studied communities: “Are you going to come and introduce this thing and then leave? Or are we going to start this and it won’t work properly? And then we're not really serving the patients very well” (CP11). Table 5 summarizes the themes across all three research questions

#### 3.4.9 Quantitative results

Cost was most often rated as an important barrier among innovation domain characteristics (42%). A third of participants rated “trialability” as important, i.e. the ability to pilot an innovation out on a small scale before scaling and undoing it if needed. A quarter rated the evidence base as important. Complexity, innovation design and adaptability (the ability to modify or tailor an innovation to fit local needs), were rated as important by relatively few participants (8–17%). See Supplementary Table 5.

Alignment with the organization’s mission was the highest rated inner setting barrier, as was the potential need to add more staff, and its compatibility with their organizations’ existing workflows and systems. A third rated physical and technical space, and the relative priority of new innovations, as important barriers. Staff training needs and training accessibility were infrequently rated as important. See Table 6.

## 4 Discussion

In this exploratory sequential mixed methods study of addiction treatment center leadership, we found that participants generally held positive attitudes toward treatment innovation, suggesting that if the field has been slow to adopt new practices, it is unlikely to be due to resistant leadership rather than organizational, workforce, and regulatory challenges (Brown & Flynn, 2002). Improving patient outcomes and retention were the most salient motivations for pursuing treatment innovations, with referral relationships and financial sustainability featuring prominently in interviews but less important on surveys. Participants’ decision-making processes largely mapped onto existing implementation frameworks, with new programs mostly originating from participants’ professional networks or clinical staff rather than academic journal articles. Participants identified philosophical incompatibility and financial barriers as the most consequential barriers to adoption. Each of these findings is discussed in more detail below.

Regarding motivations to adopt innovations, consistent with Knudsen and Roman (2015), improving patient outcomes and retention were frequently mentioned in interviews and highly rated in surveys, suggesting that facility leaders were committed to improving patients’ well-being as a first priority. Improving referral relationships was also prominent in interviews and consistent with Knudsen and Roman (2015), yet surprisingly ranked among the lowest-rated survey motivations. Attracting more *patients* was rated highly, instead. This discrepancy may reflect that participants viewed patient outcomes and positive business outcomes (more referrals, attracting more patients to their facilities) as closely linked, and may have rated “improving patient outcomes” as the highest-priority motivation to reflect that. Similarly, improving staff morale was a highly-rated motivation in surveys, despite rarely being mentioned in interviews, suggesting that facility leaders believe that adopting new programs may actually reduce staff burnout. This is consistent with past studies (Knudsen & Roman, 2004) and suggests that allowing staff to pursue clinical interests could benefit morale. Together, these findings suggest that patient outcomes, business sustainability, and staff well-being are less competing priorities than mutually reinforcing expressions of the same underlying goal.

Regarding decision-making processes, our findings generally aligned with the EPIS framework (Aarons et al., 2011), and offered further details about how addiction treatment facilities approach each stage differently from other healthcare facilities. Our results support the notion that few in treatment settings first learn about new approaches to treatment through academic journals (Gabbay & May, 2004), which is the primary way research on treatment innovations is disseminated (Nieuwland et al., 2024). Instead, facility leaders most often relied on conferences, peer networks, or from clinical staff. Once sourced, most participants reported at least consulting with various stakeholder groups, such as patients, families, and external advisors. Most also reported completing at least some structured financial analysis. Half reported piloting innovations before implementing at scale. Together, these findings suggest that, in contrast with the field’s reputation for informal decision-making (Marsch et al., 2020), addiction treatment facilities often follow a more deliberate and structured process for adopting and implementing new approaches to treatment.

Regarding barriers to innovation, our qualitative findings generally aligned with past implementation research (Geerligs et al., 2018), with participants suggesting that relative priority, high staff workloads, staff skepticism, cost, internal bureaucracy, the need for additional staff training, and regulatory burdens all may be important barriers. Quantitative findings helped prioritize these, with the need to add staff and physical and technical infrastructure among the most highly-rated concerns. Consistent with past studies (Knudsen & Roman, 2015; McGovern et al., 2004), philosophical incompatibility with an organization's treatment mission was among the most significant barriers, with medication services frequently hindered by 12-step or abstinence-only orientations. Our results were novel in identifying the potential role that workflow incompatibility could play in hindering adoption of addiction innovations. Similarly, although past studies have emphasized the importance of “trialability” (Overbye-Thompson & Hamilton, 2025), our results are among the first to affirm its importance to innovation in addiction. Cost, staffing requirements, philosophical misalignment, and regulatory complexity were the most consequential barriers, with workflow incompatibility and limited trialability emerging as underappreciated and understudied obstacles.

These findings have several practical implications for designers and disseminators of treatment innovations in addiction care. First, developers should evaluate their products’ impact on patient outcomes, but also consider how they might attract new patients, strengthen referral relationships, and reduce staff burnout. Second, dissemination efforts should prioritize professional conferences and influential clinical networks. Clinical staff may be especially influential partners, since they are frequently the source of innovation ideas that reach executive decision-makers. Developers should invest in understanding how patients, families, and community members perceive their services, since facility leaders consult these groups when making adoption decisions. Fourth, innovations should be designed with trialability in mind, meaning that facilities can pilot them at small scale, evaluate their impact, and reverse course if needed without significant cost or disruption. Finally, workflow compatibility should be treated as a design requirement rather than an afterthought, since philosophical and operational misalignment was among the most consequential barriers facility leaders identified.

### 4.1 Limitations

Several limitations are important to note. First, this sample was small: only 12 participants in total. Results should be interpreted as preliminary and hypothesis-generating. Future research should collect similar survey data in larger samples. Second, our sample included only facility leaders from the northeastern US. Results could be different among similar participants located elsewhere in the US. Finally, our sample included primarily non-Hispanic White men. Although approximately 60% of those we initially contacted to participate were women, our final sample reflects those who ultimately agreed to participate and scheduled an interview.

### 4.2 Summary

In summary, addiction treatment facility leaders held generally positive attitudes toward innovation and followed relatively deliberate adoption processes, with patient outcomes, business sustainability, and staff wellbeing functioning as mutually reinforcing rather than competing motivations. Philosophical misalignment, cost, and regulatory complexity emerged as the most consequential barriers, while workflow incompatibility and limited trialability were identified as underappreciated obstacles. Future research should replicate and extend these findings in larger, more geographically diverse samples, and should explore whether motivations, decision-making processes, and perceived barriers differ meaningfully between executive leaders and clinical directors.

## Supplementary Material

Supplementary Files

This is a list of supplementary files associated with this preprint. Click to download.

- SupplementaryTables.docx

## Figures and Tables

**Table 1:** Organizational, workforce, and intervention-related factors that are associated with adoption of addiction treatment innovations

Category	Determinant	Innovation Type	Study
***Facilitators***			
Organizational	Larger organization size	Medications	Abraham & Roman (2010)
Organizational	Higher *%* of patients with private insurance	Medications	Abraham & Roman (2010)
Organizational	Use of similar EBPs (high “innovation compatibility”)	Medications	Abraham & Roman (2010)
Organizational	History of research involvement	CM / General	Bride et al. (2011); Ducharme et al. (2011)
Organizational	Outpatient-only setting	CM / Behavioral	Bride et al. (2011); Ducharme et al. (2011)
Organizational	Diverse revenue sources	General	Ducharme et al. (2011)
Organizational	Serving specific populations (e.g., drug court)	CM	Bride et al. (2011)
Workforce	High staff education (% with Master's degrees)	General	Henggeler et al. (2008); Knudsen & Roman (2004)
Workforce	Engages in “environmental scanning”	General	Knudsen & Roman (2004)
Organizational	Surveys payer feedback	General	Knudsen & Roman (2004)
Workforce	Positive staff attitudes toward innovation	Behavioral	Henggeler et al. (2008)
Organizational	Consistency with facility's treatment philosophy	General	Knudsen & Roman (2015)
Organizational	Capacity to improve the facility's reputation	General	Knudsen & Roman (2015)
Organizational	Capacity to reduce patient dropout rate	General	Knudsen & Roman (2015)
**Barriers**			
Organizational	For-profit status	CM	Ducharme et al. (2011)
Organizational	Organizational stress / complex client needs	Behavioral	Lundgren et al. (2012)
Organizational	Long organizational history (institutional inertia)	General	Lundgren et al. (2012)
Organizational, workforce	Strong 12-step clinical orientation	Medications	McGovern et al. (2004)
Workforce	Staff turnover & workforce instability	GM / General	Becker et al. (2003) Miller et al. (2006)
Workforce	Competing clinical priorities	Behavioral	Henggeler et al. (2008)
Organizational	Limited access to expertise or prescribing physicians	Medications	Abraham & Roman (2010)
Organizational	Lack of knowledge or funding	General	Amodeo et al. (2011); Abraham & Roman (2010
Intervention	High implementation costs	Medications	Abraham & Roman (2010)
Workforce	Client or staff resistance	Behavioral	Amodeo et al. (2011), Henggeler et al. (2008)
Intervention	Burdensome certification processes	Behavioral	Amodeo et al. (2011)

**Table 2:** Demographic and occupational characteristics of treatment facility leaders (*N* = 12)

Characteristics	
	Mean (*SD*) or *N* (%)
Age (Range: 33–65)	49.2 (10.3)
Gender	
Woman	2 (15.4)
Man	11 (84.6)
Ethnicity (Hispanic or Latino)	2 (15.4)
Race	
White	13 (100.0)
Black or African American	0 (0.0)
Asian	0 (0.0)
American Indian/Alaska Native	0 (0.0)
Multiracial	0 (0.0)
Education	
College grad	2 (15.4)
Graduate or professional degree	9 (69.2)
Doctorate degree (e.g., PhD, PsyD, EdD)	1 (7.7)
Medical doctorate (e.g., MD, DO)	1 (7.7)
Position	
Chief executive officer, executive director, or president	8 (66.7)
Clinical director	4 (33.3)

**Table 3:** Characteristics of participants’ organizations, as reported by participants (*N* = 12)

Characteristics	
	Mean (*SD*) or *N* (%)
Government-owned	0 (0.0)
Type	
For-profit, freestanding	8 (66.7)
Not-for-profit, freestanding	2 (15.4)
Hospital or hospital-affiliated	2 (15.4)
Member of a provider association^1^	6 (50.0)
Accredited^2^	11 (91.2)
Services	
Inpatient detox	5 (41.7)
Residential program	5 (41.7)
Partial hospital program	5 (41.7)
Intensive outpatient program	8 (66.7)
Outpatient treatment	11 (91.7)
Recovery residences or communities	2 (16.7)
Recovery coaching (peer-to-peer support)	2 (16.7)
Multiple locations	4 (33.3)
Treatment philosophy	
12-Step model	3.3 (1.7)
Cognitive behavioral	4.2 (1.3)
Medical/psychiatric	3.9 (1.0)
Spiritual counseling	2.5 (1.1)
Facility years in operation	34 (40.7)
Number of patients, total^3^	1,200 (5,200)
Percent of patients with SUD by type	
Alcohol	44.8 (22.9)
Opioids	28.6 (13.4)
Stimulants	7.6 (6.2)
Cannabis	6.5 (5.9)
Other/polydrug	12.4 (12.4)
Number of staff, total^3^	300 (335)
Number of counselors, total^3^	16.5 (87.5)
Number of staff supervised^4^	15.4 (14.4)
Percent of staff with Master's degrees	56.4 (29.0)
Percent of staff who are licensed	53.1 (31.9)
Physician on staff	
Yes, physician (MD, DO)	10 (83.3)
Yes, mid-level provider (nurse practitioner, physician assistant)	2 (16.7)
Physician work status	
On staff	9 (75.0)
On contract	3 (25.0)
Surveys referrers?	
No	2 (16.7)
Yes, at least once	4 (33.3)
Yes, periodically	6 (50.0)
Surveys payers?	
No	5 (41.7)
Yes, periodically	7 (58.3)

Note.

1 Provider associations referred to facility-level professional membership organizations that represent ad support substance use treatment providers by offering training, providing advocacy, and certifications (e.g., National Association of Addiction Treatment Providers or the American Association for the Treatment of Opioid Dependence).

2 Refers to whether or not facilities were accredited by a recognized accrediting authority, such as the Joint Commission or the Council on Accreditation.

3 Reported as median (IQR) because of significant range.

4 Assessed among clinical directors only.

**Table 4:** Treatment executives’ motivations for considering innovations in treatment (*N = 12)*

Motivations	*Mdn*	*IQR*	*Prop. of non-zero* ^1^
To improve your patients' well-being	22	40	100.0
To attract more patients to your facility	10	12	75.0
To improve staff morale, satisfaction, or burnout, or help recruit new staff	10	11	75.0
To comply with new accreditation standards	7	19	66.7
To differentiate your facility from competitors	5	13	66.7
To be perceived as an innovative treatment facility	2	10	58.3
To improve your leverage in contracts with payers	0	16	50.0
To meet reporting or quality metrics	0	10	50.0
To serve underserved or marginalized populations	0	10	50.0
To align with the organization's mission	0	10	41.7
To align with state or federal funding priorities	0	5	33.3
To fulfill a personal commitment to improving the field	0	6	33.3
To attract more referrals (from doctors, courts, insurers)	0	0	25.0
Pressure from the board, investors, or peer organizations	0	0	16.7

Note.

1 Indicates the proportion of participants who responded to each item with something other than zero.

**Table 5:** Summary of qualitative themes by research question.

Research Question	Theme
*Q1: Motivations*	3.2.1 Improving patient outcomes and retention
	3.2.2 Improving referral relationships and reputation
	3.2.3 Financial sustainability
	3.2.4 Organizational mission
	3.2.5 Staff fulfillment and morale
*Q2: Decision-making process*	3.3.1 Exploration: Triggered by internal problem recognition more than external evidence
	3.3.2 Exploration: Structurally constrained by operational bandwidth
	3.3.3 Preparation: Intensity scales with innovation complexity, not organizational policy
	3.3.4 Preparation: Financial vetting is deliberately informal and oriented toward sufficiency rather than return
	3.3.5 Implementation: Piloting is the starting point, and implementation is inherently iterative
	3.3.6 Implementation: Clinician ownership is both a facilitator of implementation and a deliberate organizational strategy
	3.3.7 Sustainment: Sustainment decisions balance clinical outcomes and financial viability, but facilities differ in how they sequence and weight these criteria
*Q3: Barriers to adoption*	3.4.1 Innovation domain: The relative priority of innovations is low compared to standard practices
	3.4.2 Innovation domain: Innovations are more likely to be adopted when they can be piloted and reversed
	3.4.3 Inner setting domain: Everyday demands are already overwhelming for staff
	3.4.4 Inner setting domain: Facility's treatment philosophy is incompatible with some innovations
	3.4.5 Inner setting domain: Internal bureaucracy, cost, and training
	3.4.6 Outer setting domain: Regulatory requirements (and conflicts between them)
	3.4.7 Outer setting domain: Reimbursement negotiations
	3.4.8 Individuals domain: Patients are sensitive to even small frictions
	3.4.9 Individuals domain: Staff skepticism

**Table 6:** Treatment facility executives’ (*N* = 11) ratings of the importance of various “Inner setting” domain barriers to adopting treatment innovations

Whether we have the physical space to provide it	*M*	*SD*	*Prop. rated* ≥ *4*^1^
	3.36	0.50	33.3
Whether we have the technology we need to provide it	3.09	0.94	33.3
Whether we would need to add more staff	3.18	1.03	41.7
Whether our existing staff would need additional training	2.64	1.02	8.3
Whether it is compatible with our workflows, systems, and processes	3.00	1.10	41.7
Whether other programs are more important	2.82	1.08	33.3
Whether it is aligned with our mission	3.27	0.90	41.7
Whether guidance or training that is needed to deliver the intervention is accessible	2.91	0.83	25.0
Whether we have the supplies to deliver it	2.82	0.78	16.7

Note.

1 Represents proportion of participants who rated a given item at least “quite important.”
